# Inhibition of miR-450b-5p ameliorates hepatic ischemia/reperfusion injury via targeting CRYAB

**DOI:** 10.1038/s41419-020-2648-0

**Published:** 2020-06-12

**Authors:** Zuotian Huang, Tong Mou, Yunhai Luo, Xingyu Pu, Junliang Pu, Lei Wan, Junhua Gong, Hang Yang, Yanyao Liu, Zhongtang Li, Ai Shen, Zhongjun Wu

**Affiliations:** 1 Department of Hepatobiliary Surgery, the First Affiliated Hospital of Chongqing Medical University, Chongqing, China; 2 West China School of Medicine, Sichuan University, Chongqing, China; 3 Phase I Clinical Trial Ward, First Affiliated Hospital of Chongqing Medical University, Chongqing, China; 4 Hepatobiliary Pancreatic Tumor Center, Chongqing University Cancer Hospital, Chongqing, China

**Keywords:** Inflammation, Liver diseases

## Abstract

Hepatic ischemia/reperfusion injury (IRI) is an unavoidable course in liver transplantation, during which the immune response of inflammation plays a leading part. MicroRNA-450b-5p (miR-450b-5p), which has been reported to participate in several inflammatory diseases, was investigated in this study. The purpose of this study is to identify the potential function of miR-450b-5p toward remission of hepatic IRI and elucidate the specific mechanism. Herein we found that expression of miR-450b-5p, interleukin (IL)-1β, tumor necrosis factor-α (TNF-α), and IL-6 was stimulated in hepatic IRI. Inhibition of miR-450b-5p could remarkably alleviate mouse hepatic IRI and improve liver function measured by hematoxylin–eosin (HE) staining, terminal deoxynucleotidyl transferase dUTP nick-end labeling (TUNEL), and enzyme-linked immunosorbent assay (ELISA). We further assessed protein expression undergoing Western blot and immunofluorescence, and discovered that miR-450b-5p suppressed alpha B-crystallin (CRYAB), thus restraining the inhibitory κB kinase (IKK) β-mediated canonical nuclear factor-κB (NF-κB) signaling, instead of the noncanonical path guided by IKKα in hepatic IRI. In addition, we demonstrated CRYAB as an activator of M2 polarization through protein kinase B (Akt) 1/mammalian target of rapamycin (mTOR), thus resulting in relief of liver IRI. Combination treatment containing both paths revealed a better antidamage efficacy than adjusting either path alone, suggesting that the joint therapy might be a promising solution in hepatic IRI.

## Introduction

Liver transplantation has gradually become one of the most effective treatments for various end-stage liver diseases, during which the hepatic ischemia/reperfusion injury (IRI) is an inevitable pathological process that can lead to liver dysfunction and graft loss^1,2^. However, the whole molecular mechanism of hepatic IRI remains to be unfolded^3,4^. Occurrence of inflammation induced by NF-κB plays a critical part among these disparate pathogeneses^5–7^. While activation of NF-κB canonical pathway results in release of inflammatory cytokines with requirement of IKKβ phosphorylation, noncanonical NF-κB demands the codependence of IKKα and NF-κB-inducing kinase (NIK)^8–11^.

As an important member of small heat-shock protein, CRYAB displays a proper role with identification of the anti-inflammatory effect^12,13^. Recent studies indicated that CRYAB could serve as a potential target in response to stimulation of inflammatory cytokines, through suppression of IKK in intestinal mucosal inflammation^14^. Given the pivotal role of IKK in the initiation of NF-κB signaling, CRYAB is likely to control the occurrence and progress of inflammation in hepatic IRI to some extent, the specific molecular mechanism of which needs an in-depth exploration^8,15^.

For the past few years, microRNAs have been testified to an essential regulator in pathology and physiology of liver^16,17^. miR-450b-5p was reported to be a latent biomarker in transient ischemic attack and liver self-healing plasmodium malaria^18,19^. In our preliminary research, miR-450b-5p was significantly increased in cell hypoxia/reoxygenation (H/R) model, which is the classical in vitro model of hepatic IRI^20^.

Hence, in the present study, we investigated the impact of miR-450b-5p on hepatic IRI through targeting CRYAB. Furthermore, we identified a unique pattern that CRYAB reduced canonical NF-κB pathway via preventing activation of IKKβ. Besides, we underscored the involvement of CRYAB in macrophage M2 polarization through Akt1/mTOR, thus allowing a full participation to its anti-inflammatory function in hepatic IRI.

## Results

### Expression of miR-450b-5p and NF-κB pathway-associated protein was increased in hepatic IRI accompanied by downregulation of CRYAB

**Fig. 1:**
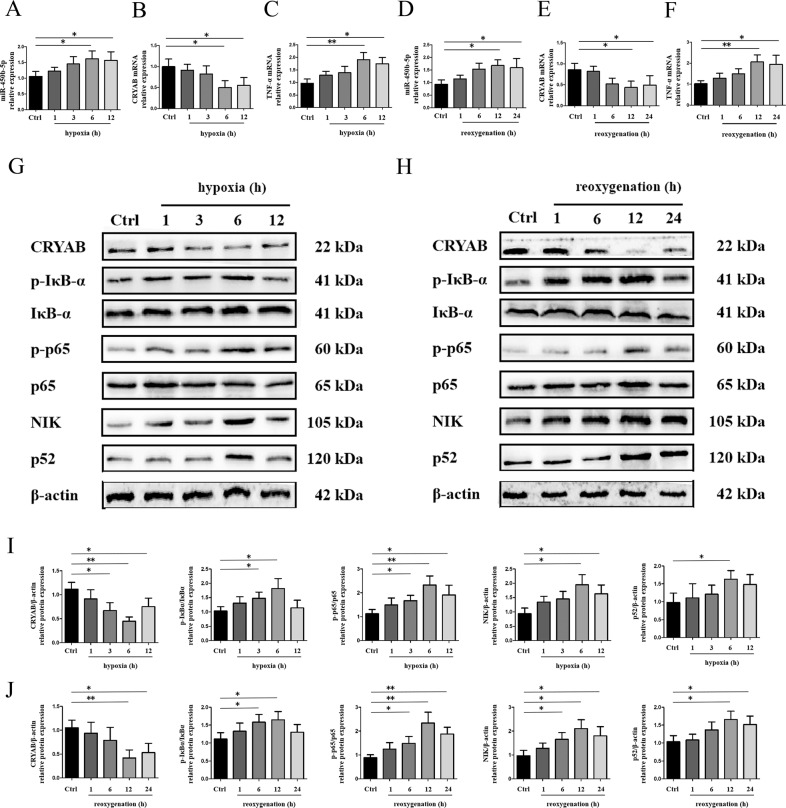
miR-450b-5p was upregulated in vitro. **a**–**c** Expression of miR-450b-5p, CRYAB, and TNF-α among hypoxia time nodes (reoxygenation for 12 h). **d**–**f** Expression of miR-450b-5p, CRYAB, and TNF-α among reoxygenation time nodes (hypoxia for 6 h). **g**, **i** Protein expression of CRYAB, p-IκBα, IκBα, p-p65, p65, NIK, and p52 among hypoxia time nodes (reoxygenation for 12 h). **h**, **j** Protein expression of CRYAB, p-IκBα, IκBα, p-p65, p65, NIK, and p52 among reoxygenation time nodes (hypoxia for 6 h). ^*^P < 0.05, ^**^P < 0.01.

H/R model as a classic in vitro model of hepatic IRI was constructed to examine the possible role of miR-450b-5p and CRYAB. RAW 264.7 cells were challenged by hypoxia within subsequent reoxygenation treatment. Time nodes were set rigorously in order to select an appropriate H/R time combination considering forepassed researches^5,21^. A persistent growth of miR-450b-5p (Fig. 1a, d) and TNF-α (Fig. 1c, f) was detected and peaked at hypoxia for 6 h, reoxygenation for 12 h in vitro. Meanwhile, both mRNA (Fig. 1b, e) and protein (Fig. 1g–j) levels of CRYAB continued to slide down within extension of H/R time. At length, hypoxia for 6 h (Fig. 1a–c) along with 12-h reoxygenation (Fig. 1d–f) was chosen for the follow-up experiments. Phosphorylation of the inhibitor of NF-κB (IκB) α and NF-κB p65 was simultaneously enhanced faced with H/R (Fig. 1g–j). Remarkably, while downstream canonical NF-κB was stimulated, there was also a significant activation of the noncanonical NF-κB pathway, scilicet the increase of NIK and NF-κB p52 (Fig. 1g–j).

### Inhibition of miR-450b-5p decreased expression of canonical NF-κB within recovery of CRYAB

**Fig. 2:**
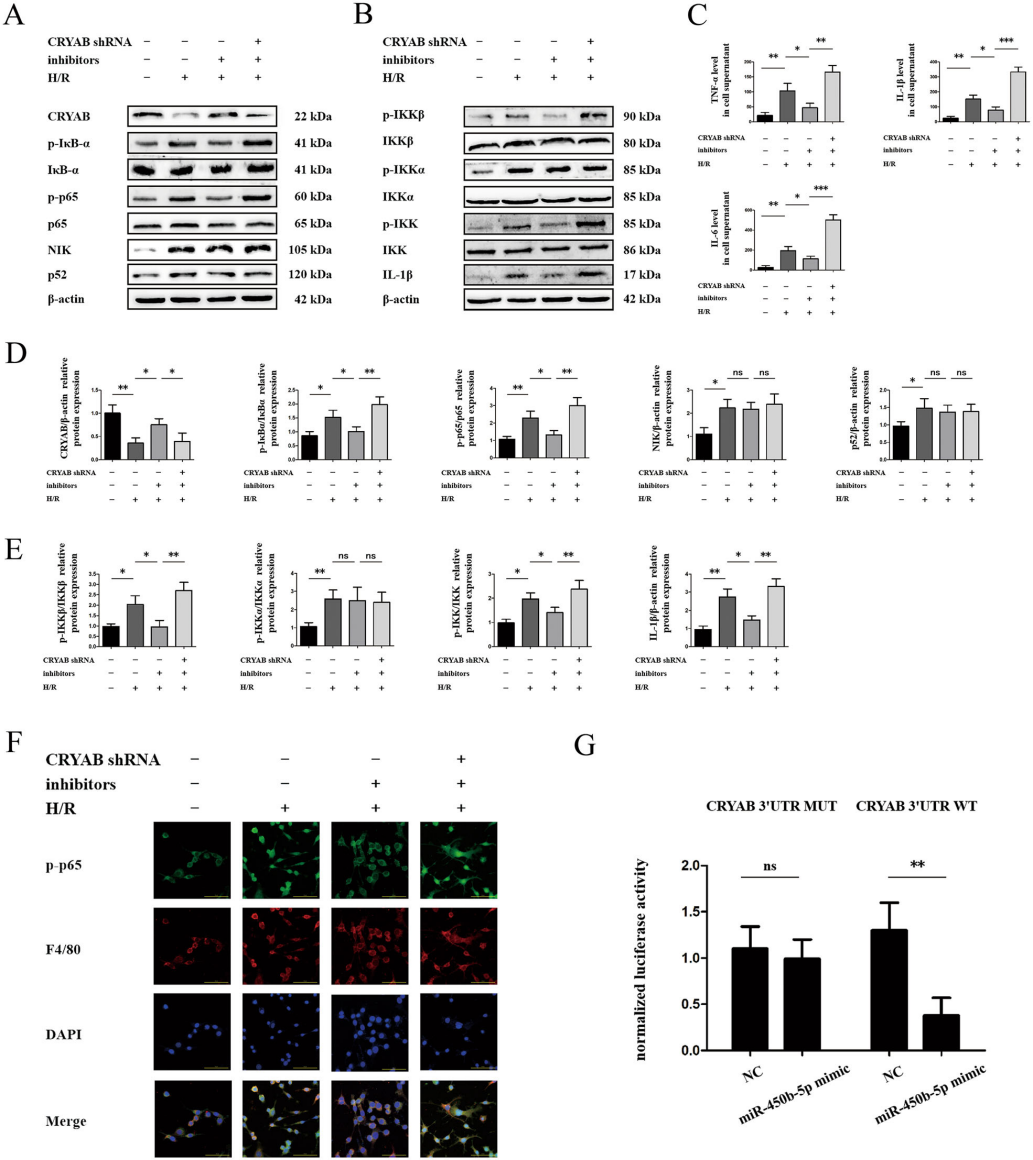
Relation of miR-450b-5p and CRYAB. **a**, **b**, **d**, **e** Protein expression of CRYAB, canonical, and noncanonical NF-κB signal-associated protein in miR-450b-5p inhibitors or CRYAB shRNA groups. **c** Levels of IL-6, IL-1β, and TNF-α in cell supernatant. **f** Immunofluorescence of p-p65 and F4/80 in miR-450b-5p inhibitors or CRYAB shRNA groups. Scale bar = 50 μm. **g** Double-luciferase reporter gene experiment of miR-450b-5p targeting CRYAB. ^*^P < 0.05, ^**^P < 0.01, ^***^P < 0.001, ^ns^*P* > 0.05.

In order to illuminate the characteristics of miR-450b-5p and CRYAB in vitro, miR-450b-5p was inhibited in the first place followed by suppression of CRYAB. As expected, miR-450b-5p inhibitors drove a reduction of pro-inflammatory protein accompanied with the augment of CRYAB (Fig. 2a, d), thereby leading to a blockade of inflammatory cytokines, including IL-6, TNF-α, and IL-1β (Fig. 2c). However, the noncanonical NF-κB pathway represented by launch of NIK and p52 protein remained unchanged after inhibition of miR-450b-5p (Fig. 2a, d). Further refraining CRYAB could regain the inflammation in miR-450b-5p-suppressive groups, and the relevant downstream participants seemed to be IKKβ/p65 instead of IKKα/p52, allowing for inaction of either NIK (Fig. 2a, d) or p-IKKα (Fig. 2b, e) faced with CRYAB alterations. The activation of canonical NF-κB pathway detected by immunofluorescence of p-p65 nuclear translocation was identified, simultaneously (Fig. 2f). Moreover, double-luciferase reporter containing wild-type (WT) or mutant (MUT) CRYAB 3′ UTR was transfected into HEK293 cells. As shown, miR-450b-5p observably inhibited luciferase activity in WT groups, whereas no significant alteration was monitored in MUT groups, indicating that miR-450b-5p directly binds to CRYAB 3′ UTR (Fig. 2g). Negative control (NC) showed no function (Fig. 2g).

### Downregulation of miR-450b-5p alleviated mouse hepatic IRI in vivo

**Fig. 3:**
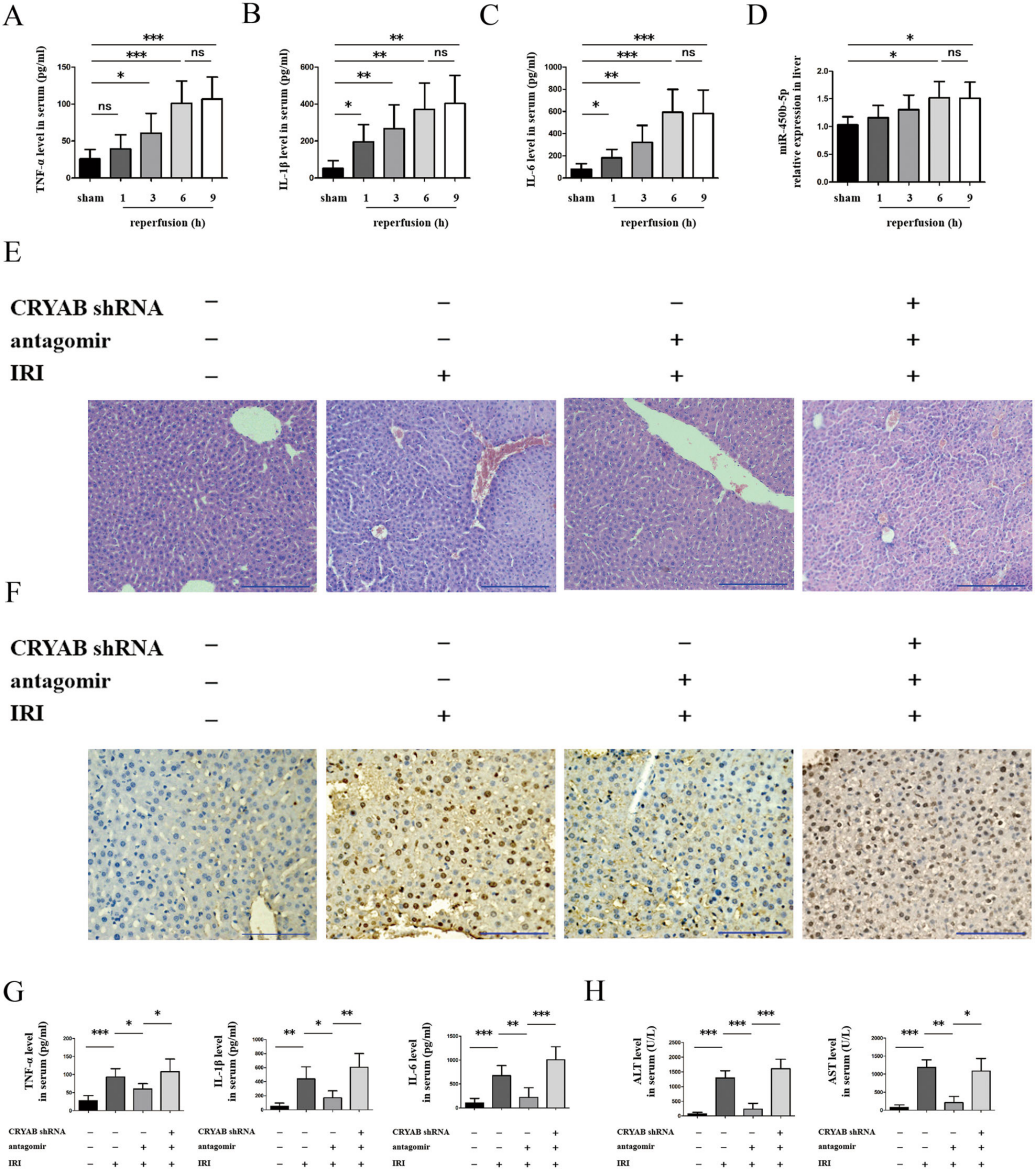
Influence of regulating miR-450b-5p and CRYAB in vivo. **a**–**c** Serum levels of IL-6, IL-1β, and TNF-α within 1-h ischemia followed by 1-, 3-, 6-, and 9-h reperfusion. **d** Expression of miR-450b-5p in the liver within 1-h ischemia followed by 1-, 3-, 6-, and 9-h reperfusion. **e** Hepatic pathologic alteration after inhibition of miR-450b-5p or CRYAB within 1-h ischemia followed by 6-h reperfusion, scale bar = 200 μm. **f** Hepatic apoptosis detected by TUNEL within 1-h ischemia followed by 6-h reperfusion, scale bar = 200 μm. **g**, **h** Levels of IL-6, IL-1β, TNF-α, AST, and ALT in mouse serum within 1-h ischemia followed by 6-h reperfusion. CRYAB shRNA-, antagomir-, and IRI group was settled as sham group. *n* = 5 for each group, ^*^P < 0.05, ^**^P < 0.01, ^***^P < 0.001, ^ns^*P* > 0.05.

Serum levels of IL-6, TNF-α, and IL-1β were augmented within 1-h ischemia followed by extension of reperfusion time (1, 3, 6, and 9 h) as shown in Fig. 3a–c. Analogously, when reperfusion time extended to 6 h, expression of miR-450b-5p in the liver peaked and had no significant difference from the 9-h group (Fig. 3d). These results indicated that combination of 1-h ischemia followed by 6-h reperfusion was appropriate for subsequent in vivo experiments. miR-450b-5p deficiency in vivo by miR-450b-5p antagomir transfection resulted in less pathophysiological changes (Fig. 3e) and apoptosis (Fig. 3f) in liver tissue. On the contrary, further interference of CRYAB could aggravate hepatic pathological injury as well as apoptosis (Fig. 3e, f). There was also a remarkable decrease of pro-inflammatory factors (Fig. 3g) and hepatic enzyme (Fig. 3h) in miR-450b-5p antagomir group, while inhibition of CRYAB rebounded this phenomenon (Fig. 3g, h).

### CRYAB restrained activation of IKKβ but had no impact on IKKα in vitro

**Fig. 4:**
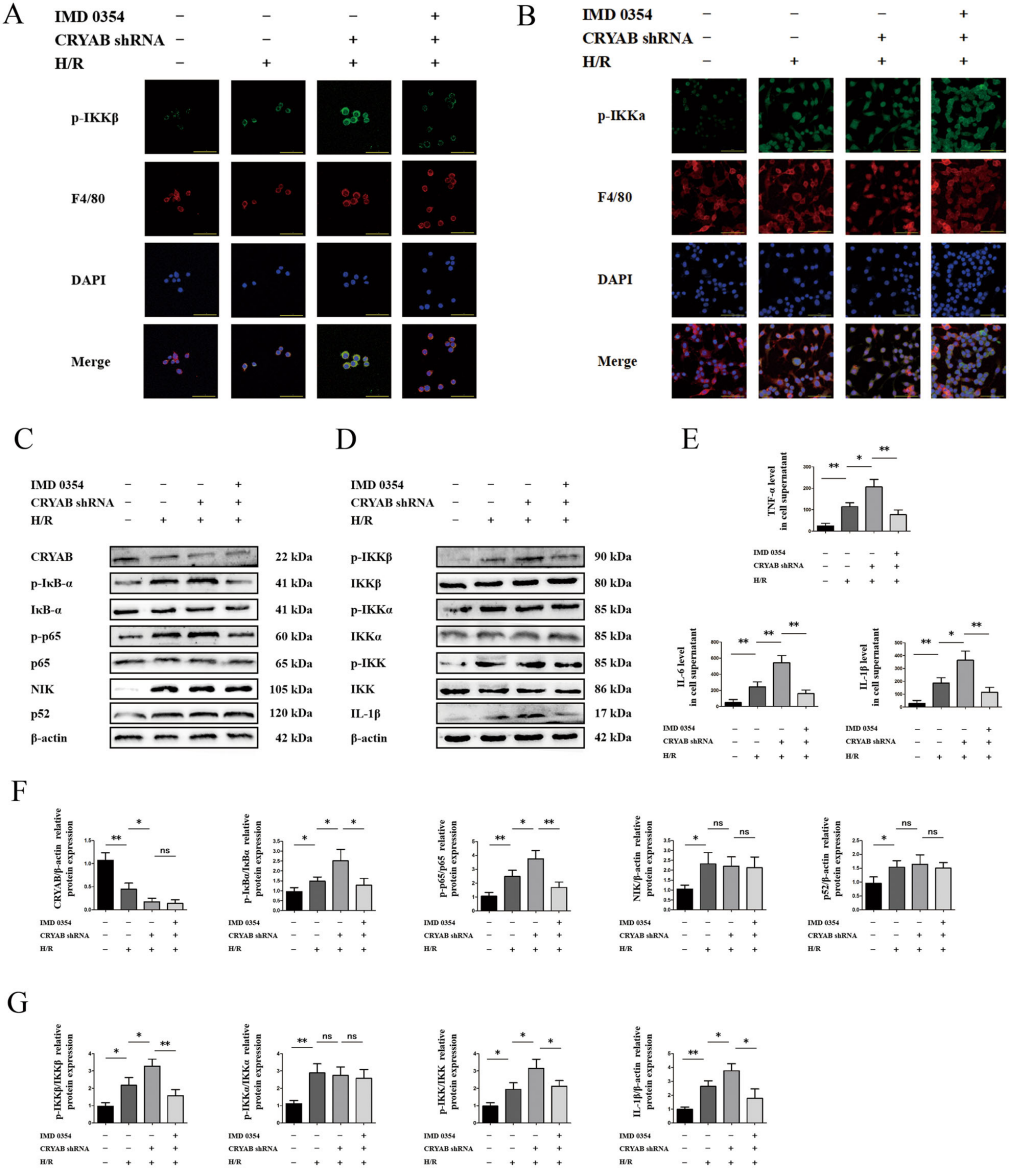
Involvement of IKKβ in CRYAB commanding canonical NF-κB pathway. **a**, **b** Immunofluorescence of p-IKKβ-F4/80 and p-IKKα-F4/80 in CRYAB knocking-down or IMD0354-utilized groups. Scale bar = 50 μm. **c**, **d, f**, **g** Impact of IMD0354 upon canonical and noncanonical NF-κB protein within CRYAB abrogation. **e** Levels of IL-6, IL-1β, and TNF-α in cell supernatant. ^*^P < 0.05, ^**^P < 0.01, ^ns^*P* > 0.05.

To confirm the relevance between IKKβ-mediating canonical NF-κB and CRYAB in hepatic IRI, IMD0354 was implemented as a classical inhibitor of IKKβ phosphorylation^22,23^. As illustrated in immunofluorescence, activation of IKKβ derived from CRYAB blockade was reversed by IMD0354 addition (Fig. 4a). There was no change in p-IKKα after either CRYAB short-hairpin RNA (shRNA) or IMD0354 modulation (Fig. 4b). Similarly, phosphorylation of both p65 (Fig. 4c, f) and IKKβ (Fig. 4d, g) was efficiently suppressed after IMD0354 treatment, which showed no effectiveness upon NIK/IKKα (Fig. 4c, d, f, g). Notably, inhibition of IKKβ could to some extent moderate the release of IL-1β, IL-6, and TNF-α induced by CRYAB shortage (Fig. 4e).

### CRYAB improved liver function in vivo partially due to IKKβ control

**Fig. 5:**
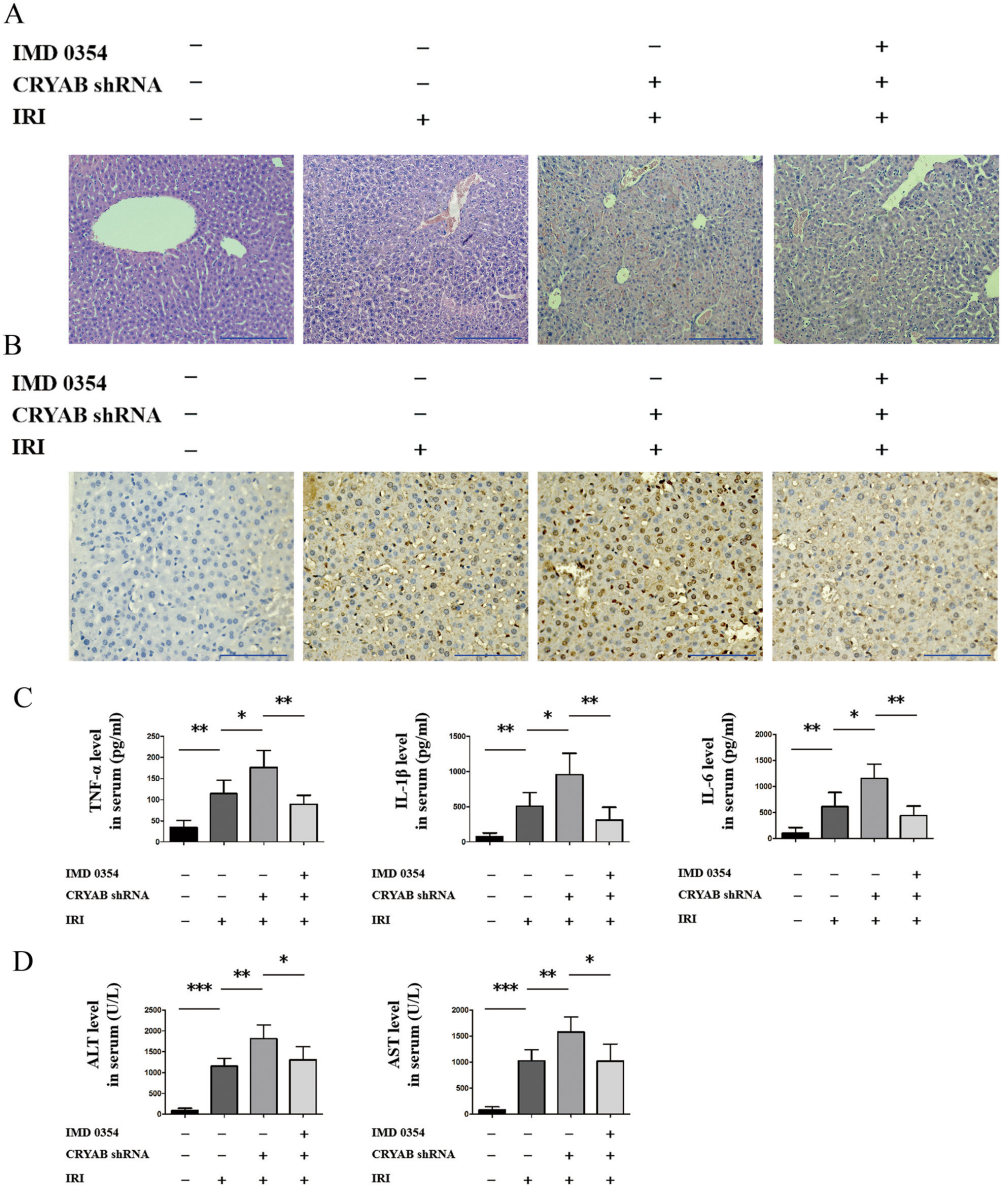
p-IKKβ scarcity partially reversed liver damage derived from CRYAB reduction after ischemia for 1 h followed by 6-h reperfusion. **a** Hepatic pathologic alteration in mouse liver after inhibition of CRYAB or IKKβ. Scale bar = 200 μm. **b** Assessment of hepatic apoptosis by TUNEL. Scale bar = 200 μm. **c** Levels of IL-6, IL-1β, and TNF-α in mouse serum. **d** Levels of AST and ALT in mouse serum. IMD 0354-, CRYAB shRNA-, and the IRI group was settled as sham group. *n* = 5 for each group, ^*^P < 0.05, ^**^P < 0.01, ^***^P < 0.001.

We further utilized IMD0354 in vivo to evaluate the function originated from IKKβ inhibition. IMD0354 was able to partially backspin the hepatic pathological lesion and apoptosis induced by CRYAB repression (Fig. 5a, b). A stimulative effect on serum IL-6, TNF-α, and IL-1β was validated in CRYAB-reduction group, while addition of IMD0354 remarkably reversed this phenomenon (Fig. 5c). In the meantime, levels of aspartate aminotransferase (AST) and alanine aminotransferase (ALT) were to some extent lower undergoing IMD0354 treatment (Fig. 5d). The antidamage effect of IMD0354 was effective but not complete, implying that some other pathway might contribute to miR-450b-5p/CRYAB axis in hepatic IRI.

### Participation of miR-450b-5p and CRYAB in M2 polarization

**Fig. 6:**
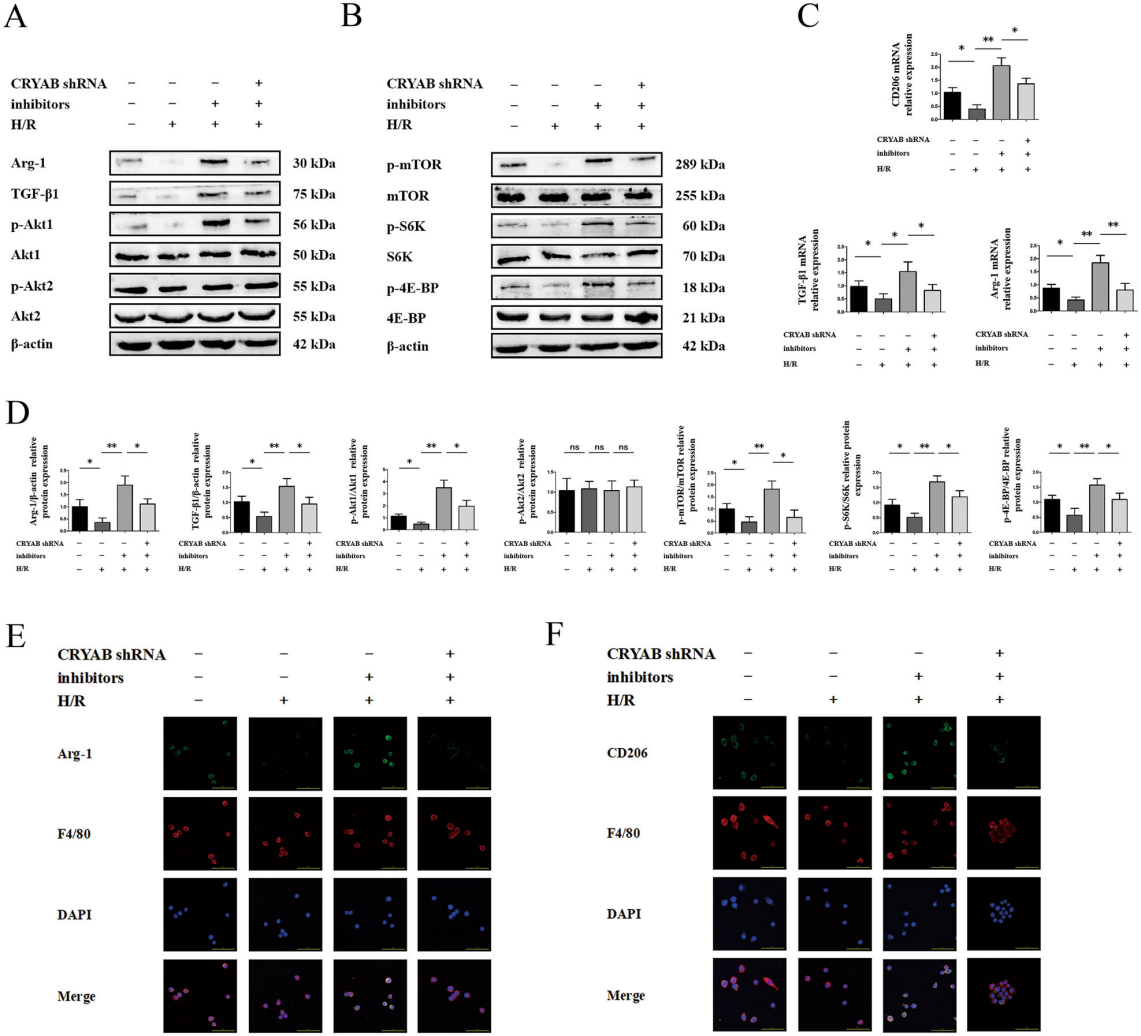
miR-450b-5p/CRYAB transformed M2 polarization. **a**, **b**, **d** Protein expression of Arg-1, TGF-β1, and Akt/mTOR pathway. **c** mRNA levels of CD206, Arg-1, and TGF-β after inhibition of miR-450b-5p or CRYAB by miR-450b-5p inhibitors or CRYAB shRNA. **e**, **f** Immunofluorescence of Arg-1 and CD206. Scale bar = 50 μm. ^*^P < 0.05, ^**^P < 0.01, ^ns^*P* > 0.05.

To verify the contribution of other potential mechanisms, we made efforts on M2 polarization considering its known immune participation in liver IRI. Expression of CD206, TGF-β1, and Arg-1 was reduced facing H/R disposition, while suppression of miR-450b-5p by miR-450b-5p inhibitors showed a facilitating effect on them (Fig. 6a–d). In contrast, interference of CRYAB blocked expression of M2 polarization-relevant markers (Fig. 6a–d). As consistent with mRNA, the results of Western blot (Fig. 6a–d) and immunofluorescence (Fig. 6e, f) confirmed that protein expression of Arg-1, TGF-β1, and CD206, and activation of Akt/mTOR pathway, were motivated using miR-450b-5p inhibitors. Meanwhile, CRYAB downregulation prevented expression of M2 polarization-related protein (Fig. 6a–d), as well as phosphorylation of Akt (Fig. 6a–d). It was worth mentioning that Akt1/mTOR, instead of Akt2, seemed to be the primary channel triggered by either miR-450b-5p or CRYAB (Fig. 6a–d).

### Trigger of Akt1/mTOR was engaged in CRYAB-inducing M2 polarization in vitro

**Fig. 7:**
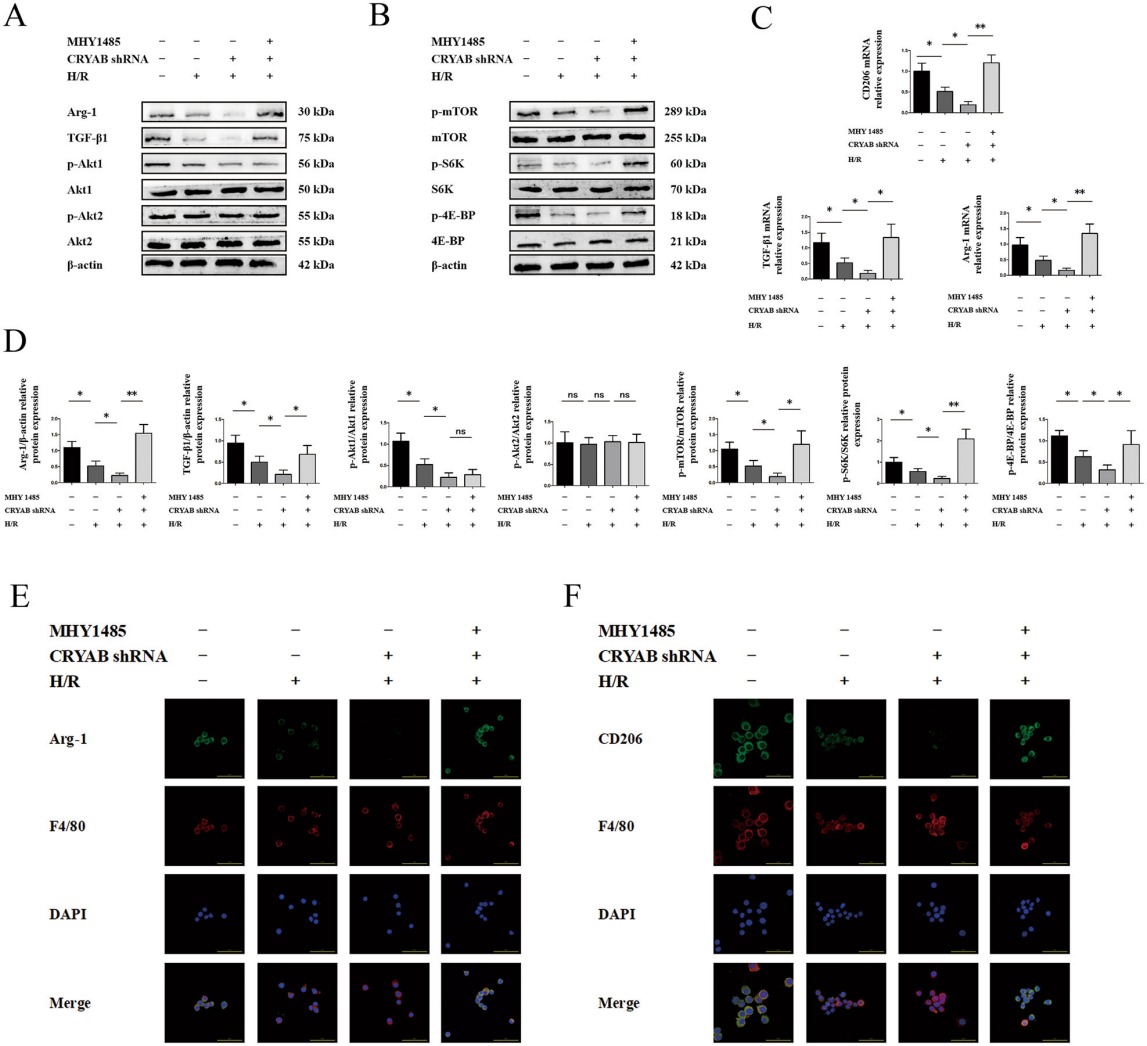
Activation of mTOR reversed M2 polarization from CRYAB reduction in vitro. **a**, **b**, **d** Protein expression of Akt/mTOR after CRYAB inhibition or MHY1485 usage. **c** mRNA levels of CD206, Arg-1, and TGF-β1. **e**, **f** Immunofluorescence of Arg-1 and CD206. Scale bar = 50 μm. ^*^P < 0.05, ^**^P < 0.01, ^***^P < 0.001, ^ns^*P* > 0.05.

A classic mTOR activator named MHY1485 was applied to validate the internal route of CRYAB commanding M2 polarization in vitro^24,25^. In CRYAB low-expression groups, M2 markers, Akt1/mTOR pathway and phosphorylation of pivotal downriver transcription factors, were declined (Fig. 7a–d). Nevertheless, entry of MHY1485 changed the immune environment by uplifting activation of mTOR (Fig. 7b–d), suggesting a major participative characteristic of Akt1/mTOR in CRYAB regulating M2 polarization. M2 markers were also rescued at both protein (Fig. 7a, b–d) and mRNA (Fig. 7c) levels in mTOR pathway-elevating groups.

### Co-adjustment of IKKβ and mTOR determined the outcome of hepatic IRI

**Fig. 8:**
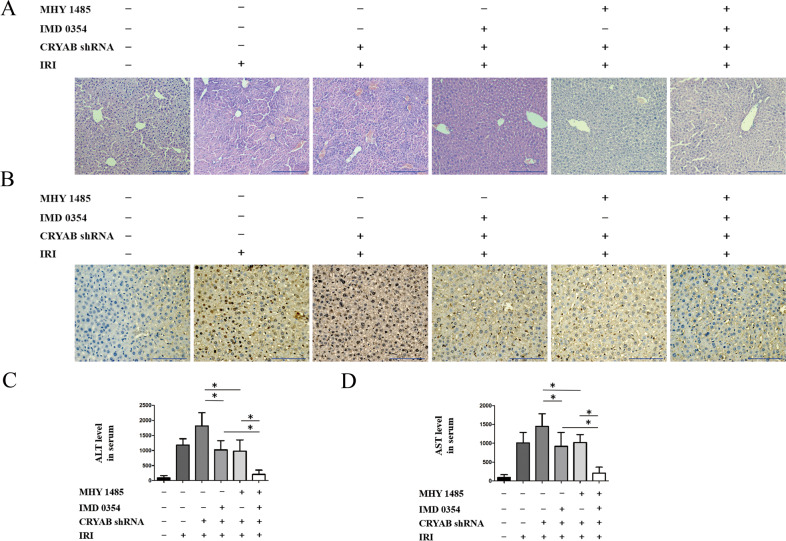
Effect of co-adjustment containing IKKβ and mTOR after 1-h ischemia followed by 6-h reperfusion. **a** Liver pathologic alteration after using IMD0354 and MHY1485 faced with IRI. Scale bar = 200 μm. **b** Detection of hepatic apoptosis, scale bar = 200 μm. **c**, **d** Release of AST and ALT in mouse serum. MHY1485-, IMD 0354-, CRYAB shRNA-, and IRI group was settled as sham group. *n* = 5 for each group, ^*^P < 0.05.

HE staining was performed to investigate the contribution of both IKKβ and mTOR in mice exposed to hepatic IRI. Either inhibition of IKKβ or activation of mTOR alone could only modulate the extent of liver injury partially (Fig. 8a). Consistent with the foregoing result, apoptosis in liver tissue was incompletely cut down because of IMD0354 or MHY1485, respectively (Fig. 8b). A co-modulation containing IKKβ suppression and mTOR ignition was constructed and consequently gave a more significant brake of liver IRI, compared with individual treatments (Fig. 8a, b). Homoplastically, IMD0354 combined with MHY1485 repressed ALT and AST in a more efficient way compared with using either method alone (Fig. 8c).

## Discussion

Hepatic IRI is a constitutive process during liver transplantation, and some hepatectomies, the mechanism of which has been so far manifested to include inflammation, oxidative stress, and apoptosis, remain not fully explored^26,27^. Development of severe inflammatory immune response as well as impaired M2 polarization could lead to uncontrolled hepatic IRI, thus resulting in poor outcome and prognosis^28,29^. In the current study, we authenticated CRYAB, restricted by miR-450b-5p, as an efficient repressor of liver IRI by dual mechanisms.

Upregulation of miR-450b-5p was validated in hepatic IRI along with downregulation of CRYAB in this study. Inhibition of miR-450b-5p could attenuate hepatic IRI both in vivo and in vitro, notably. Consistent with our findings, a previous study has identified miR-450b-5p as a potential biomarker and therapy target in brain-transient ischemic attacks^19^. Afterward, we selected out hypoxia for 6 h followed by 12-h reoxygenation for subsequent in vitro experiments on the basis of quantitative real-time polymerase chain reaction (qRT-PCR) and Western blot results in RAW 264.7 cell line, a classic surrogate of Kupffer cell, regarding the dominant position of liver-resident macrophage upon progression of hepatic IRI-induced inflammation. We then observed an accumulation of CRYAB when miR-450b-5p was impaired, so we blocked CRYAB, thus in turn rescuing the influence of miR-450b-5p inhibition. Herein, we conducted a double-luciferase gene report and verify CRYAB as a direct target of miR-450b-5p, indicating that miR-450b-5p might function in hepatic IRI at least partially through targeting CRYAB. What we found was consistent with the prediction using Targetscan7.2 that miR-450b-5p directly targets CRYAB. Recent evidences suggested that CRYAB was a protective regulator in various pathological and physiological conditions, such as neuroinflammatory injury, muscle-developing, and brain edema^30–32^. In animal study, we screened out 1-h ischemia followed by 6-h reperfusion according to accumulation tendency of both cytokines and miR-450b-5p. Knocking down of CRYAB in vivo could abolish the relieving of liver damage derived from obstruction of miR-450b-5p. It is worth noting that activation of canonical NF-κB pathway mediated by IKKβ showed relevance to CRYAB blockade, while NIK/IKKα remained invariant. What we testified was consistent with previous studies, in which CRYAB was proved to be a potent negative regulator of noncanonical NF-κB signal^14^.

We further introduced a particular IKKβ suppressant, IMD0354, for in-depth inquiry about the connection between CRYAB and IKKβ in hepatic IRI. IMD0354 was reported to mitigate peripheral and corneal inflammation^33,34^. Similar to these researches, IKKβ-selective erasing by IMD0354 obtained an ideal anti-inflammatory effect in our study. However, it was remarkable that IKKβ inhibition could only in part, but not in whole, lead to remission of hepatic IRI caused by CRYAB shortage. This phenomenon demonstrated that some other pathway might contribute to CRYAB modulation in hepatic IRI. Based on that, our vision shifted onto the relevance between CRYAB and mTOR pathway regarding the critical role of M2 polarization in organ IRI^35,36^.

An irritant of H/R was capable of restraining M2 polarization in view of M2 marker, including CD206, Arg-1, and TGF-β1, showing a coherence with previous studies^37,38^. We noticed a noteworthy M2 phenotype with additional miR-450b-5p inhibition on the basis of H/R, while further interference of CRYAB could weaken this appearance. Downstream from CRYAB, we revealed that Akt1/mTOR was involved in miR-450b-5p/CRYAB axis-mediated M2 polarization. The ability of CRYAB-administering Akt1 signal was formerly proved in another model^39^, and our findings gained an in-depth view in respect to the mTOR pathway. We inputted MHY1485 as a mTOR activator to distinguish the connection between CRYAB and mTOR in M2 immunophenotype favoring^40,41^. Addition of mTOR activator liberated a backspin on M2 polarization, in which side the effectiveness of CRYAB knocking down was disabled, indicating that CRYAB regulated immunological competence of macrophage in hepatic IRI, besides the immune consequence of inflammation itself, in a mTOR-dependent manner.

There was a certified relation between CRYAB and M2 polarization in non-small-cell lung cancer^42^, while our results made an appraisal of CRYAB as a M2 phenotype ignitor thus participating in hepatic IRI. In consideration of CRYAB adjusting both IKKβ and mTOR, we established a co-accommodation containing IKKβ constraint and mTOR excitation, by using IMD0354 and MHY1485 together. Single use of either treatment could not entirely recover CRYAB shortage-derived liver IRI, while co-accommodation led to a complete remission to some extent.

In conclusion, we found that miR-450b-5p directly targeted CRYAB in hepatic IRI. The capacity of CRYAB comprised dual mechanisms, both IKKβ-inducing canonical inflammatory cascade and Akt1/mTOR-mediating preparation of macrophage overactivating. Our findings disclosed a possible biomarker and target in hepatic IRI. In addition, joint regulation of multiple pathways might be a potential strategy in the aspect of hepatic IRI therapy.

## Materials and methods

### Cell culture and H/R model

RAW 264.7 cells were used as a classic surrogate of Kupffer cells^43,44^ and purchased from Cell Bank of the Type Culture Collection of the Chinese Academy of Sciences. All cells were incubated using Dulbecco’s Modified Eagle Medium—Nutrient Mixture F-12 (Gibco, Grand Island, USA), with a 10% concentration of fetal bovine serum (Gibco, Australia). Cell culture medium contains 100 U/ml penicillin and 0.1 mg/ml streptomycin (Beyotime, Shanghai, China). Conventional cell incubator (Thermo, MA, USA) was set into 5% CO_2_ at the temperature of 37 °C.

H/R model was employed as a classical in vitro model for hepatic IRI. Preconditioning of hypoxia was applied through a tri-gas incubator (Thermo, MA, USA) and atmospheric environment was set with N_2_ (94%), O_2_ (1%), and CO_2_ (5%). Time nodes were established cautiously for an appropriate H/R combination. Hypoxia for 1, 3, 6, and 12 h and reoxygenation for 1, 6, 12, and 24 h were attempted, respectively. Finally, 6-h hypoxia followed by 12-h reoxygenation was selected as an optimum node for subsequent experiments.

### qRT-PCR assay

Trizol (Takara, Dalian, China) was used for the extraction of total RNA according to the manufacturer’s instructions. miR-450b-5p was normalized to U6^45^, while regular mRNA was normalized to β-actin. Stem-loop miRNA First Strand cDNA Synthesis kit (Sangon Biotech, Shanghai, China) was used for cDNA synthesis of miRNA. Quantification of both miRNA and mRNA was measured using SYBP Premix Ex Taq (Takara, Tokyo, Japan). Primer sequences were obtained from Sangon Biotech (Shanghai, China) and listed in Table 1. All specimens were run in triplicate and analyzed by the 2^−ΔΔCt^ computing method.

**Table 1:** The sequences.

Name	Sequence
miR-450b-5p forward	CGCGTTTTGCAGTATGTTCC
miR-450b-5p reverse	AGTGCAGGGTCCGAGGTATT
U6 forward	AGAGAAGATTAGCATGGCCCCTG
U6 reverse	ATCCAGTGCAGGGTCCGAGG
CRYAB forward	GAGTCTGACCTCTTCTCAACAG
CRYAB reverse	AGAACCTTGACTTTGAGTTCCT
CD206 forward	GGAATCAAGGGCACAGAGTTA
CD206 reverse	ATTGTGGAGCAGATGGAA
TNF-α forward	TATGGCTCAGGGTCCAACTC
TNF-α reverse	GGAAAGCCCATTTGAGTCCT
Arg-1 forward	CTGCCTGCTTTCTGAGTGCTGAG
Arg-1 reverse	CCTGTGGTTCCGATAAGTGCTTCC
TGF-β1 forward	AGGGCTACCATGCCAACTTC
TGF-β1 reverse	CCACGTAGTAGACGATGGGC
β-actin forward	GGCTGTATTCCCCTCCATCG
β-actin reverse	CCAGTTGGTAACAATGCCATGT
miR-450b-5p mimic forward	UUUUGCAGUAUGUUCCUGAAUA
miR-450b-5p mimic reverse	UUCAGGAACAUACUGCAAAAUU
miR-450b-5p inhibitors	UAUUCAGGAACAUACUGCAAAA
miR-450b-5p antagomir	UAUUCAGGAACAUACUGCAAAA
CRYAB shRNA (target sequence)	GGAACTCAAAGTCAAGGTTCT

### Western blot and immunofluorescence procedure

Protein extraction and quantification procedure of CRYAB (CST, Shanghai, China), p-IκBα, IκBα, S6K, 4E-BP (Santa Cruz Bio, Santa Cruz, USA), IKKα, IKKβ, p-p65, p65, IKK, p-IKK, Arg-1, TGF-β1, β-actin, and p-4E-BP (Beyotime, Shanghai, China), IL-1β (Wanleibio, Shanghai, China), NIK, p52, p-IKKα, p-IKKβ (Invitrogen, CA, USA), p-Akt1, Akt1, p-Akt2, Akt2, p-mTOR, mTOR, and p-S6K (Abcam, Cambridge, UK) was consistent with our previous researches^6,46^. Primary antibody dilutions for Western blot are listed in Table 2.

**Table 2:** Antibody dilutions for Western blot.

Antibody name	Dilution
Akt1, mTOR	1/5000
p-Akt1, p-p65, p-IκBα, IκBα, and p-mTOR	1/2500
p52, β-actin	1/2000
S6K	1/1500
CRYAB, NIK, p65, IKKα, p-IKKα, p-IKKβ, IKKβ,	
IKK, p-IKK, IL-1β, Akt2, p-Akt2, Arg-1, and p-4E-BP	1/1000
p-S6K	1/800
4E-BP	1/500

Glass coverslips were used to grow cells for immunofluorescence. After H/R treatment and transfection, cells were immobilized with paraformaldehyde (4%) for 15 min. Goat serum was used for 1-h blockade. Triton X-100 (0.3% dissolved in goat serum) was used for half an hour cellular permeabilization. After incubation of anti-p-p65, F4/80, p-IKKβ (Abcam, Cambridge, UK), p-IKKα, Arg-1, and CD206 (Invitrogen, CA, USA) overnight at 4 °C, coverslips were washed softly and then reincubated with Goat anti-rabbit (FITC-labeled) or Goat anti-mouse (Cy3-labeled, Beyotime, Shanghai, China) immunoglobulin G (IgG) for 1 h, strictly avoiding light at 37 °C. Nucleus was dying by 4′,6-diamidino-2-phenylindole (DAPI) for 8 min. Primary antibody dilutions for immunofluorescence were set as follows: p-IKKα, 1:250; p-p65, p-IKKβ, and CD206, 1:200; F4/80, 1:100; observation of immunofluorescence was achieved using a laser confocal microscopy (Olympus, Tokyo, Japan).

### Cytokine and hepatic aminotransferase detection

Release of IL-6, IL-1β, and TNF-α in both cell and serum was detected by ELISA kits (Neobioscience, Beijing, China) according to the specification guide book. Detection of serum ALT and AST release was executed by liver enzyme kits (JianCheng Bioengineering Institute, Nanjing, China) following the specification guide book. Quantification of the aforesaid sample absorbance was fulfilled using a microplate reader (Biotek, Vermont, USA).

### Transfection of cells

miR-450b-5p inhibitors or NC (Genepharma, Shanghai, China) were transfected to cells 48 h before hypoxia, at a concentration of 200 nM using Lipofectamine 6000 (Beyotime, Shanghai, China). Culture fluid was replaced 6 h after transfection. CRYAB-specific shRNA and NC contained in lentivirus vectors were prepared and incubated following the manufacturer’s instructions (Genepharma, Shanghai, China). Potency of miR-450b-5p inhibitors and CRYAB shRNA was testified. IMD0354 (MedChemExpress, Shanghai, China) served as a specific IKKβ abolisher and was incubated (10 μM) with cells for 24 h prior to H/R treatment in accordance with explanatory memorandum. MHY1485 (APExBIO, Boston, USA) served as a mTOR activator and was incubated (5 μM) with cells for 6 h prior to H/R. Sequences of miR-450b-5p inhibitors and CRYAB shRNA are listed in Table 1.

### Transfection of mice

miR-450b-5p antagomir or NC (Genepharma, Shanghai, China) were prepared and injected through tail vein (10 mg/kg) 24 h before ischemia, on the basis of the specification guide. CRYAB shRNA was transfected to mice 14 days prior to IRI through tail-vein injection. The transfection efficacy of animals was verified. IMD0354 was injected into mouse enterocoelia 24 h prior to IRI, in a dose of 5 mg/kg body weight, while MHY1485 was injected intraperitoneally 6 h prior to IRI in a dose of 2.5 mg/kg body weight, according to the instructions and previous studies. Sequences of miR-450b-5p antagomir are listed in Table 1.

### Luciferase reporter system

A pmirGLO Dual-Luciferase miRNA Target Expression Vector (Promega, Madison, WI) containing WT or MUT CRYAB 3′-UTR fragments was constructed. CRYAB 3′-UTR was cloned into the XbaI site of pmirGLO vector. Luciferase reporter plasmid (80 ng), thymidine kinase promoter—Renilla luciferase reporter plasmid (40 ng), and miR-450b-5p mimic or NC (20 nm, Genepharma, Shanghai, China) were co-transfected into HEK293 cells using lipofectamine 2000 (Invitrogen, CA, USA). Sequences of miR-450b-5p mimic are listed in Table 1. A Dual-Glo Luciferase Reporter Assay System (Promega, Madison, WI) was applied for detection of Firefly and Renilla luciferase. Relative luciferase activity was calculated for binding intensity.

### Animals and IRI model

All animal experiments and related procedures were approved by the Animal Care and Use Committee, Chongqing Medical University. All male C57BL/6J mice were from Chongqing Medical University Laboratory Animals Center. Free approach to water and food was provided, and a normative circumstance with a standard 12-h dark/light cycle, temperature, and humidity was maintained. All mice aged 6–8 weeks and weighed 25–28 g, and were randomly assigned to each group. In the present study, standard of blinding and randomization was complied with.

Liver IRI model was constructed in accordance with our previous studies^6,47^. Reperfusion for 1, 3, 6, and 9 h was performed after 1-h ischemia with vascular clamping as previously depicted. Serum and liver tissues were harvested after IRI disposition.

### HE staining and TUNEL

Liver sections were fixed with paraformaldehyde. After embedding, tissues were sliced up followed by staining. Hepatic IRI pathological impairment was assessed by Suzuki score according to HE results. Hepatic apoptosis was determined using TUNEL kit (Beyotime, Shanghai, China) in line with the manufacturer’s instructions. A specific microscope (Olympus, Tokyo, Japan) and ZEN2012 were applied for observation at different magnifications.

### Statistical analysis

All in vitro experiments were repeated at least three times. As for in vivo experiments, sample size for each group was illuminated in the corresponding position, respectively, and equaled to at least five. GraphPad Prism (version 5.0, San Diego, USA) was used for data analysis. All experiment data were presented as mean ± standard error. Student’s *t* test for two groups and one-way ANOVA among groups were used to determine the significance of comparisons. A value of *p* less than 0.05 was considered to be significant.
